# Recurrent Gastrointestinal Hemorrhage in Children with Philadelphia-Positive B-Cell Acute Lymphoblastic Leukemia Treated with Dasatinib: Case Reports

**DOI:** 10.1155/2020/5678210

**Published:** 2020-02-10

**Authors:** Xin Liao, Yuxia Guo, Yali Shen, Jianwen Xiao

**Affiliations:** ^1^Department of Hematology, Children's Hospital of Chongqing Medical University, Chongqing 400014, China; ^2^Ministry of Education Key Laboratory of Child Development and Disorders, Chongqing 400014, China; ^3^China International Science and Technology Cooperation Center for Child Development and Disorders, Chongqing 400014, China; ^4^Chongqing Key Laboratory of Pediatrics, Chongqing 400014, China

## Abstract

Dasatinib, a second-line tyrosine kinase inhibitor (TKI), has been widely used in chronic myeloid leukemia (CML) and Philadelphia-positive B-cell acute lymphoblastic leukemia (Ph + B-ALL). Although dasatinib has been well tolerated, side effects including hemorrhage are not rare. Cases of bleeding disorders ultimately result in thrombocytopenia, but platelet aggregation dysfunction induced by dasatinib has also been demonstrated in Ph + B-ALL and CML patients. We report three Chinese children with Ph + B-ALL who received a combination treatment of chemotherapy and dasatinib and developed gastrointestinal bleeding several months later. The platelet count and clotting tests were normal, and these patients presented with dasatinib-induced platelet dysfunction. These findings reveal that physicians should be aware of and carefully monitor for side effects, including bleeding disorders.

## 1. Introduction

Acute lymphoblastic leukemia (ALL) is the most common cancer in children, and precursor B-cell lymphoblastic leukemia (B-ALL) accounts for 70–75% of pediatric ALL cases [1]. Pediatric B-ALL with Philadelphia positivity (Ph + B-ALL) is relatively rare and accounts for less than 5% of childhood cases of B-ALL [2]. Historically, in both children and adults, Ph + B-ALL has been considered to have the worst prognosis of all B-ALL subtypes [2]. Since the introduction of tyrosine kinase inhibitors (TKIs), the outcome for Ph^+^ B-ALL has gradually improved, and with a combination therapy of chemotherapy and TKIs, the 5-year event-free survival (EFS) rate of such patients has exceeded 80% [3]. Studies of Ph + B-ALL have revealed that the efficacy of dasatinib is superior to that of imatinib [4, 5], and the utilization of dasatinib in Ph + B-ALL is gradually increasing. Although TKIs were designed to target the fusion protein BCR-ABL1, they have shown some off-target inhibition of other tyrosine kinases [3], and it is important to recognize and manage adverse events caused by TKIs that share pathways in other organs.

In this article, we report three Ph + B-ALL children treated with dasatinib in which recurrent gastrointestinal (GI) hemorrhage was observed.

## 2. Case Reports

Three male patients were diagnosed with Ph + B-ALL between November 2016 and January 2018 at the Children's Hospital of Chongqing Medical University (CHCMU). The clinical characteristics and laboratory findings were collected and are listed in Table 1. Bone marrow (BM) samples were collected, and the diagnosis of Ph + B-ALL was confirmed according to WHO-2016 criteria [6]. The initial diagnosis of Ph + B-ALL was based on the FAB morphological classification detected by cytomorphological observation in the BM smear and biopsy. The immunophenotype was detected by flow cytometry (FCM) per protocol. The chromosomal karyotype was determined, and fluorescence in situ hybridization (FISH) was used to detect chromosomal translocations reported in the literature, including ETV6-RUNX1, MLL rearrangements, BCR-ABL1, C-MYC rearrangements, and PDGFRB rearrangements [7–9]. In total, 29 common fusion genes [10], including ETV6-RUNX1, MLL rearrangements, BCR-ABL1, TCF3-PBX1, and 27 Ph-like ALL fusion genes [11], were detected by multiplex nested reverse transcription polymerase chain reaction (multiplex RT-PCR), and positive BCR-ABL1 status was confirmed by real-time quantitative RT-PCR (qRT-PCR) [4].

Patients with Ph + B-ALL were treated as the intermediate risk group according to the CCCG-ALL-2015 protocol (the clinical trial began in 2015, registration number: ChiCTR-IPR-14005706). The protocol was divided into 4 phases: remission induction, consolidation, continuation, and maintenance. TKIs, including imatinib (300 mg/m^2^ daily, CTTQ PHARMA, China) and dasatinib (60 mg/m^2^ daily, CTTQ PHARMA, China), were administered randomly once the BCR-ABL1 fusion gene was confirmed. The three enrolled patients were administered imatinib, and treatment was suspended when the absolute neutrophil count (ANC) was <0.5 × 10^9^/L or severe infection occurred.

BM samples were obtained at different time points (TPs) throughout the duration of chemotherapy, and minimal residual disease (MRD) levels were monitored by FCM [12] at TP1 and TP2 (day 19 and day 46 of remission induction, respectively). FISH and qRT-PCR for BCR-ABL1 [13] were also performed at TP1 and TP2, and molecular remission monitored by qRT-PCR was achieved at TP2 for these patients. In the subsequent chemotherapy, FISH and qRT-PCR for BCR-ABL1 and mutation of ABL1 [13] were performed every six months, and the results were negative (Table 2).

The results of the clinical trial showed that the effect of dasatinib was superior to that of imatinib for the EFS rate (data have not been published), and the three patients were treated with a combination of dasatinib and chemotherapy instead of imatinib and chemotherapy. Changes in the type of TKI being administered occurred in the three patients at 24, 10, and 8 months after the initial administration of imatinib. The patients were admitted with consistent hemafecia and/or anemia 4 and 5 months later, when blood, white blood cells, and occult blood appeared in the stool, but stool cultures were negative. CMV and EBV were screened by PCR, and the results were negative. Platelet count, clotting tests (thrombin time, prothrombin time, international normalized ratio of prothrombin time, activated partial thromboplastin time, fibrinogen, and D-dimer), and coagulation factor (VII, VIII, IX, XI, XIII, and vW factor) tests were normal; platelet aggregation tests induced by adenosine diphosphate (ADP) were performed, and the results were abnormal (Table 3).

**Figure 1:**
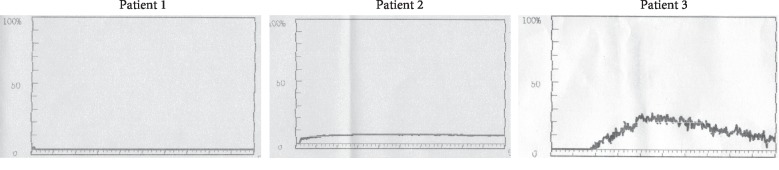
PLT function tests of the three patients.

**Figure 2:**
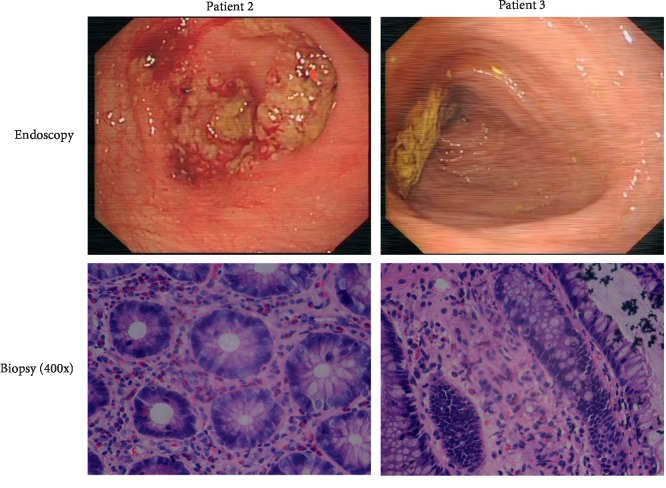
Endoscopy and biopsy results of the two patients.

Endoscope and biopsy were refused by patient 1, and examinations were performed on patient 2 and patient 3. Patient 2 presented with ulcerative colitis under endoscopy, and infiltration of lymphocytes and eosinophils was detected under a microscope. Patient 3 presented with normal colonic mucosa under endoscopy, but eosinophilic infiltration was also found by histopathology. These data are shown in Figures 1 and 2.

Concerning comedications such as nonsteroidal anti-inflammatory drugs (NSAIDs), glucocorticoids, and traditional herbs were not administered before GI bleeding, and the side effects leading to hemafecia were suspected to be caused by chemotherapy and/or dasatinib. Chemotherapy was continued when GI hemorrhage vanished, and hemafecia did not reappear. Patients presented with hemafecia once dasatinib had been administered for 2–4 weeks even when the initial dosage of dasatinib was reduced to 20 mg daily.

Dasatinib-induced GI hemorrhage in the Ph + B-ALL patients was confirmed. Chemotherapy was continued, and imatinib was carefully increased from a dosage of 100 mg/m^2^ daily to 300 mg/m^2^ daily; hemafecia disappeared. The patients remained in complete remission and underwent chemotherapy and imatinib treatment.

## 3. Discussion

Ph + B-ALL is a subtype of B-ALL in which blast cells are detected to have a translocation between BCR on chromosome 22 and the ABL1 oncogene on chromosome 9; this molecular subtype accounts for 2–4% of pediatric ALL cases and approximately 20% of adult ALL cases [1–4]. TKIs have been approved for the treatment of chronic myeloid leukemia (CML) and Ph + ALL [1], and the prognosis of Ph + B-ALL has improved since the introduction of combination treatments with TKIs and chemotherapy [2–5]. Imatinib and dasatinib are the most widely used TKIs in the Ph + B-ALL population.

Dasatinib had 325-fold greater potency than imatinib in cells transduced with the BCR-ABL fusion gene [2]. In addition, outstanding outcomes with the combination of dasatinib and chemotherapy were confirmed in a clinical trial, and the three patients in this report received dasatinib instead of imatinib. TKIs, including dasatinib, have several side effects, and toxicities occur in many organs and/or systems [14]. The majority of side effects induced by TKIs involve the hematological and endocrine systems.

With the widespread use of TKIs, bleeding complications induced by dasatinib have been reported in chronic myeloid leukemia patients [15–17] and Ph + B-ALL patients [18–20]. Adverse bleeding events have been reported in 26–40% of patients with CML or Ph + ALL who received dasatinib [2–4]. The majority of bleeding occurred in the GI tract, and the main reason for bleeding was thrombocytopenia due to myelosuppression or hematopoietic cell transplantation [19, 21], whereas GI hemorrhage in such patients with a normal platelet count is uncommon.

In this article, the patients presented with dasatinib-induced GI hemorrhage, and we think that bleeding was mainly due to abnormal platelet aggregation. It is well known that damaged platelet function leads to bleeding, whereas proteins targeted by dasatinib are implicated in the dynamic processes of platelet activation, likely contributing to the bleeding diathesis seen with dasatinib use [22]. Comedications that may lead to GI bleeding are common in leukemia patients [23], familiar drugs including NSAIDs, glucocorticoids, and traditional Chinese herbs had not been prescribed to the patients at least 2 weeks before the GI bleeding, and thus the role of comedications in the GI bleeding was excluded. Patient 2 had ulcerative colitis, which is uncommon during dasatinib treatment [24], and the underlying pathophysiology remains poorly understood. Dasatinib shows some off-target inhibition of other tyrosine kinases [3], and platelet-derived growth factor receptor (PDGFR) kinase is a potent target of dasatinib [14]. It has been reported that PDGFR-null mice have defective angiogenesis and capillary wall development, which lead to microaneurysm formation and hemorrhage [15].

In summary, we report three Chinese children with Ph + B-ALL who received a combination therapy of TKIs and chemotherapy. They presented with GI hemorrhage due to the side effects of dasatinib; GI hemorrhage disappeared when they were administered with imatinib. These cases reveal that platelet function should be monitored in patients who receive dasatinib.

## Figures and Tables

**Table 1:** Clinical characteristics and laboratory findings of the patients.

Clinical and laboratory findings	Patient 1	Patient 2	Patient 3
Age at diagnosis (m)	115	64	141
Clinical presentation	Bone pain, adenopathy, and hepatosplenomegaly	Fever, pale appearance, adenopathy, and hepatosplenomegaly	Pale appearance, ecchymosis, headache, adenopathy, and hepatosplenomegaly
WBC count (×10^9^/L)	107.21	299.36	223.39
PLT count (×10^9^/L)	140	20	22
Hb level (g/L)	126	58	88
Blasts in PB	0.86	0.95	0.89
BM smear	ALL-L_2_	ALL-L_2_	ALL-L_2_
BM biopsy	B-ALL	B-ALL	B-ALL
Immunophenotype	Common B-ALL	Common B-ALL	Common B-ALL
Chromosomal karyotype	45, XY, −7, *t*(9;22)(q34;q11)	45, XY, −20, *t*(9;22)(q34;q11)	45, XY, −7, *t*(9;22)(q34;q11)
FISH for BCR-ABL1	(+), 90%	(+), 96%	(+), 85%
Fusion gene screening	BCR-ABL1(P210)	BCR-ABL1(P190)	BCR-ABL1(P210)
IS BCR-ABL1	1.049	0.743	0.126

WBC: white blood cell; PLT: platelet; Hb: hemoglobin; PB: peripheral blood; BM: bone marrow; FISH: fluorescence in situ hybridization.

**Table 2:** Results of MRD, FISH, and qRT-PCR analyses for the patients.

Time point	Laboratory findings	Patient 1	Patient 2	Patient 3
TP1	MRD	<10^−4^	0.38%	1.38%
	FISH	Negative	Negative	Negative
	IS BCR-ABL1	0.012	0.015	0.0015
TP2	MRD	<10^−4^	<10^−4^	<10^−4^
	FISH	Negative	Negative	Negative
	IS BCR-ABL1	Negative	Negative	Negative
Every 6 months after chemotherapy	FISH	Negative	Negative	Negative
	IS BCR-ABL1	Negative	Negative	Negative
	ABL1 mutation	Negative	Negative	Negative

TP: time point; MRD: minimal residual disease; FISH: fluorescence in situ hybridization.

**Table 3:** Laboratory findings for hemafecia status.

Results	Patient 1	Patient 2	Patient 3
Hb level (g/L)	127	127	70
PLT count (×10^9^/L)	206	255	197
Clotting tests	Normal	Normal	Normal
PLT function	Abnormal	Abnormal	Abnormal
Stool test	RBC (4+), WBC (+), OB (+)	RBC (5+), WBC (2+), OB (+)	RBC (2+), WBC (+), OB (+)

PLT: platelet; Hb: hemoglobin; ND: no data.
